# Practical Consequences of the Bias in the Laplace Approximation to Marginal Likelihood for Hierarchical Models

**DOI:** 10.3390/e27030289

**Published:** 2025-03-11

**Authors:** Subhash R. Lele, C. George Glen, José Miguel Ponciano

**Affiliations:** 1 Department of Mathematical and Statistical Sciences, University of Alberta, Edmonton, AB T6G 2R3, Canada; 2 Archie Carr Center for Sea Turtle Research, Gainesville, FL 114000, USA; george.glen@ufl.edu; 3 Department of Biology, University of Florida, Gainesville, FL 114000, USA; josemi@ufl.edu

**Keywords:** biased estimating functions, data cloning, errors in variables, Markov Chain Monte Carlo

## Abstract

Due to the high dimensional integration over latent variables, computing marginal likelihood and posterior distributions for the parameters of a general hierarchical model is a difficult task. The Markov Chain Monte Carlo (MCMC) algorithms are commonly used to approximate the posterior distributions. These algorithms, though effective, are computationally intensive and can be slow for large, complex models. As an alternative to the MCMC approach, the Laplace approximation (LA) has been successfully used to obtain fast and accurate approximations to the posterior mean and other derived quantities related to the posterior distribution. In the last couple of decades, LA has also been used to approximate the marginal likelihood function and the posterior distribution. In this paper, we show that the bias in the Laplace approximation to the marginal likelihood has substantial practical consequences.

## 1. Introduction

Hierarchical models are extremely useful for modeling complex phenomena. For the theory and applications of this class of models, see [1,2,3,4], among the plethora of books and papers. This class of models, however, has an Achilles heel. The likelihood function for the parameters of these models involves very high dimensional integration over the unobserved or latent variables. The advent of the Markov Chain Monte Carlo (MCMC) algorithms [5,6] provided an ingenious but computationally intensive solution. These computational difficulties can be circumvented, at least in some situations, by using Laplace’s method to approximate an integral. Both [7,8] used the Laplace approximation to obtain computationally fast and accurate values for posterior means and other quantities. The Laplace approximation works under the condition that the likelihood function can be approximated by a quadratic function. This is usually the case, at least for regular models [9]. However, the Tierney and Kadane method is applicable only when the likelihood function can be expressed analytically, which is often not the case for general hierarchical models.

A simulation-based approach for approximating the likelihood function, without assuming that it is nearly quadratic, was developed by Geyer and Thompson ([10]). The Geyer–Thompson approach has also been used to obtain the profile likelihood function ([11,12]) for a function of the parameters. MCMC type algorithms can be used to compute the maximum likelihood estimator and its asymptotic variance without computing the likelihood function explicitly using Data Cloning ([13,14]), Monte Carlo Newton Raphson (MCNR) or Monte Carlo Expectation Maximization (MCEM) ([15]). Reference [16] used data doubling ([17]), in conjunction with data cloning to compute the profile likelihood function for an arbitrary function of the parameters. These methods, however, are computationally intensive.

In the context of Normal Linear Mixed Models (NLMM), the Henderson method III ([4]) replaces the latent variables by their conditional expectations given the observations. Behaving as if these are the true values of the latent variables, one can express the likelihood function explicitly and conduct the standard likelihood-based inferences. The advantage of this method is that it completely avoids the messy and difficult multidimensional integration. This method was extended to the Generalized Linear Mixed Models (GLMM) by [18]; however, ref. [19] raised a cautionary note about it. The h-likelihood proposed by [20] follows up on Henderson’s method III for general hierarchical models where the latent variables are replaced by the mode of the conditional distribution of the latent variables given the observed variables. The method of h-likelihood works in some situations ([21]) but not in general. The main problem with the replacement of latent variables by their ‘estimates’, as was pointed out by various discussants of [20] paper, is that information about latent variables does not necessarily increase with an increase in the sample size. This is the classic Neyman–Scott problem ([22]) where the number of unknowns increases at the same rate as the number of observations. In a famous paper, Skaug and Fournier [23] assume that the conditional distribution of the latent variables, given the observed variables themselves, can be approximated by the Laplace approximation. Under this assumption, they propose a computationally fast method to obtain an approximation to the marginal likelihood function. See also [24,25] for computational details and applications. The profile likelihood function for any function of the parameters can be computed from the Skaug–Fournier approximation by using constrained optimization. A similar approximation was used by [26] in their development of the popular Integrated Nested Laplace Approximation (INLA) method to compute fast and accurate approximations to various posterior quantities when the distribution of the latent variables have a specific structure of a Gaussian Markov Random Field. Interestingly, in the discussion of the paper by [26] a number of discussants noted the connection to h-likelihood and questioned the validity of INLA.

In this paper, we take another look at the Laplace approximation used in [23,26]. We show that the resultant approximation is, in general, a biased approximation. This result is not surprising and was anticipated previously but was not formalized. This result was recently formalized by [27]. However, Han and Lee [27] emphasize the development of an alternative to the LAML and do not emphasize the important practical consequences of this result. Without such an explanation with practical examples, one can dismiss the result by claiming that “the bias could be small, hence perhaps one can ignore it in practice”. Indeed, many of the review papers and books about LAML, even knowing the caveats raised by the discussants of Lee and Nelder [20], seem to make this argument by using simulation studies that LAML is a good enough approximation of the marginal likelihood. Our paper dispels this argument by showing that the consequences of the bias in LAML are practically significant. Indeed, the Laplace approximation can produce confidently wrong answers that can easily go unnoticed by practitioners using off-the-shelf packages that are widely used and recommended [28].

In this paper, we show that the bias does not converge to zero as the sample size increases. This can lead to estimators that are asymptotically biased and inconsistent. Moreover, the Hessian matrix for such an approximation also tends to be different than the Hessian matrix of the true marginal likelihood. Hence, asymptotic confidence intervals also tend to have incorrect coverage; in fact, coverage probability can converge to zero as the sample size increases. We illustrate these issues using simple linear and generalized linear regression models where covariates are measured with error. Given the immense popularity of statistical packages, e.g., glmmTMB and TMB, that are based on this approximation, these results, at the minimum, suggest that before applied scientists use this technique in complex situations, a careful theoretical exploration of the limitations of the Laplace approximations to the marginal likelihood is needed.

## 2. Laplace Approximation of the Marginal Likelihood Function for Hierarchical Models

We note that the following mathematical result was anticipated by a few discussants of [20,26]. See also [21]. Unbeknownst to us, this result was recently formalized by [27].

We roughly follow the notation and description in [23].

***Notation:***

Hierarchy 1: $Y|U=u∼f(y|u,\theta_{1})$

Hierarchy 2: $U∼g(u;\theta_{2})$

Let $\theta =(\theta_{1},\theta_{2})$ be a parameter vector. The joint distribution of (Y,U) is as follows: f(y,u;θ)=f(y|u;θ)g(u;θ)=f(y;θ)h(u|y;θ). Recall that, when considered as a function of θ, f(y;θ)=L(θ;y) is the marginal likelihood function. The Laplace approximation of the marginal likelihood function (LAML) is obtained as follows:

1. Maximize f(y,u;θ) with respect to *u* for a fixed value of (y,θ). Let us denote this value by $\hat{u}\left(y,\theta \right)$ or, with a slight abuse of notation, by $\hat{u}=\underset{u}{argmax}logf\left(y,u;\theta \right)$. Let $H\left(\hat{u}\right)=\frac{\partial^{2}}{\partial^{2}u}logf\left(y,u;\theta \right){|}_{u=\hat{u}}$ denote the Hessian matrix, the matrix of second derivatives, at the location of the maximum. Notice that it is implicitly assumed that logf(y,u;θ) as a function of *u*, for every fixed value of (y,θ), has a unique maximum and is differentiable as a function of *u*. Hence, it is required that all latent variables are continuous random variables.
2. Then $L^{*}\left(\theta ;y\right)=f\left(y,\hat{u};\theta \right)\times {\left|H\left(\hat{u}\right)\right|}^{-0.5}$ is the Laplace approximation to the marginal likelihood ([23] Equation (3)).
3. The profile likelihood function for any function of the parameters ψ(θ) can be computed by using any constrained optimization routine, namely: $PL\left(\psi ;y\right)=\underset{\theta :\psi (\theta )=\psi }{argmax}L^{*}\left(\theta ;y\right)$.

We rewrite the Laplace Approximation to the marginal likelihood function (LAML) as follows: $L^{*}\left(\theta ;y\right)=f\left(y,\hat{u};\theta \right)\times |H\left(\hat{u}\right){|}^{-0.5}=L\left(\theta ;y\right)f\left(\hat{u}|y;\theta \right)\times {\left|H\left(\hat{u}\right)\right|}^{-0.5}.$ It is clear that LAML is appropriate provided

(1) $$\int f\left(u|y,\theta \right)du\approx f\left(\hat{u}|y;\theta \right)\times {\left|H\left(\hat{u}\right)\right|}^{-0.5}\approx 1$$

We note that Equation (1) has to hold true for all values of the parameter θ and for every sample *y*. In general, $f\left(\hat{u}|y;\theta \right)\times {\left|H\left(\hat{u}\right)\right|}^{-0.5}$ varies with θ, for a fixed value of *y*. The question then is under what conditions would this be equal to 1 for all values of (θ,y)?

1. Equation (1) will be exactly true provided logf(u|y;θ) is a quadratic function of *u*. For example, if U|Y=y follows a Gaussian distribution for all values of (y,θ). Notice that for the LMM, this is assumed, and hence, Henderson’s method III works.
2. Equation (1) might hold true if the number of random effects is fixed or if they increase at a slower rate than the rate at which the sample size increases. For example, in the multi-stratum studies, if the number of strata is m=o(n) (e.g., [22,29]) where *n* is the sample size and *m* is the number of strata. In this case, information about the random effects *u* in the data *y* increases to infinity at an appropriate rate. Then, the conditional distribution U|Y=y is likely to converge to a Gaussian distribution as the sample size increases. Hence, at least asymptotically, the Laplace approximation may be good.
3. In most other situations, unless explicitly demonstrated, $f\left(\hat{u}|y;\theta \right)\times {\left|H\left(\hat{u}\right)\right|}^{-0.5}$ is of order O(n) (e.g., [26], Section 4.1). In general, $f\left(\hat{u}|y;\theta \right)\times {\left|H\left(\hat{u}\right)\right|}^{-0.5}$ varies with θ. An immediate consequence is that the score function, the first derivative with respect to θ based on the LAML even asymptotically, is not a zero unbiased estimating function ([21,30,31]). An immediate consequence of the biased estimating function is that the resultant estimators are inconsistent.
4. The fact that $f\left(\hat{u}|y;\theta \right)\times {\left|H\left(\hat{u}\right)\right|}^{-0.5}$ varies with θ also affects the second derivative matrix, and the resultant estimating function is not information unbiased ([21,31]). The lack of information unbiasedness implies that statistical inferences such as the confidence intervals or the likelihood ratio tests based on the Laplace approximated marginal and profile likelihood function are likely to be misleading.
5. Equation (1) clearly fails if the conditional distribution f(u|y,θ) is not unimodal. Establishing unimodality of the conditional distribution f(u|y,θ) is difficult in practice. For example, consider models where the dimension of the latent variables *U* is larger than the dimension of the observations *Y*. In such a situation, there are possibly several values of *U* that are compatible with an observed value of Y=y. Hence, the conditional distribution is likely to be multimodal and LAML would not be applicable.

It was pointed out by various discussants of [20] that in most practical situations, the information in the observations about latent variables is limited. When the distribution f(u|y,θ) is unimodal, the precise quantification of what we mean by ‘sufficient’ information is given by the bias factor $f\left(\hat{u}|y;\theta \right)\times {\left|H\left(\hat{u}\right)\right|}^{-0.5}$. If the bias factor, as sample size increases, converges to a constant for all parameter values and for every sample, we have sufficient information; otherwise, we do not. Unfortunately, this bias factor cannot be computed for general hierarchical models without resorting to the MCMC algorithm. Hence, we cannot easily judge in practice if the Laplace Approximation to the Marginal Likelihood (LAML) is valid.

If the conditional distribution f(u|y,θ) is multi-modal, its Laplace approximation is trivially invalid. The multimodality can possibly be checked in practice by using a multitude of starting values when computing $\hat{u}$ in Step 1 in the computation of LAML. If different starting values lead to different LAML estimates, one should be wary of using the methodology.

Let us look at the LAML as a penalized or regularized likelihood where the penalty function is $log\left(f\left(\hat{u}(y,\theta \right)|y,\theta \right)|H\left(\hat{u}(y,\theta \right){\left|y,\theta )\right|}^{-0.5})$. We do not know how this penalty behaves as a function of θ for general hierarchical models. If this is a multimodal function of θ, it can potentially lead to $L^{*}\left(\theta ;y\right)$ being multimodal even when the true likelihood, L(θ;y), is unimodal. Even when the penalty function is unimodal but with the mode located away from the mode of the true likelihood, the mode of the LAML will be located away from the true MLE. The second derivative of the penalty function, with respect to the parameter, is non-zero. Thus, the Hessian matrix based on $L^{*}\left(\theta ;y\right)$ cannot be used to compute asymptotic variance or asymptotic confidence intervals. This penalty function also affects the value of the likelihood function at the location of the mode. In practice, this value is used to conduct likelihood ratio tests or model selection using various information criteria. We illustrate in the next section how misleading this penalty function can be for computing the MLE and for computing the profile likelihood for a parameter of interest.

The following examples show that even for simple yet important statistical models, the ‘bias’ factor, $f\left(\hat{u}|y;\theta \right)\times {\left|H\left(\hat{u}\right)\right|}^{-0.5}$, is not constant and the LAML fails to approximate the true likelihood function. On the other hand, for the same models, the MCMC-based approaches work quite well. We also present two simple examples where the conditional distribution f(u|y,θ) is multimodal and LAML, as well as MCMC-based approaches, fail to approximate the true likelihood function.

## 3. Counter Examples

We now present examples where the Laplace approximated marginal likelihood (LAML) function is a bad approximation to the true marginal likelihood function. In these examples, the location of the maximum of the LAML does not coincide with the location of the maximum of the true likelihood, even for large sample sizes. As a consequence, for large enough samples, the confidence intervals based on the LAML end up missing the true value entirely. It also appears that the curvature of the LAML can be quite different than the curvature of the true likelihood function.

The Geyer–Thompson (GT) approximation ([10,12]) is an alternative method to compute an approximation to the true likelihood. This is based on the MCMC algorithm and hence is computationally intensive. GT does not assume anything about the shape of the likelihood function. However, it involves choosing a reference value of θ. Different choices of the reference value can potentially lead to different answers. It is suggested ([10]) that multiple reference values be chosen and the results averaged to obtain a reliable approximation. Using multiple reference values, however, can increase the computational load substantially. In our experience, if the MLE is chosen as the reference value, the GT approximation appears to work well. However, it does not always properly capture the curvature of the likelihood function at the location of the maximum. Thus, GT can lead to inappropriate confidence intervals.

The DC, MCNR and MCEM algorithms can be used for computing the MLE and its associated Fisher Information matrix without computing the likelihood function explicitly. Data cloning followed by the Data Doubling method (DCDD) can be used to compute the profile likelihood function. It seems to work reasonably well in most situations.

Notice that similar to [7], DCDD only assumes that logL(θ;y) is approximately quadratic as a function of θ, at least for large sample sizes. This holds true for most regular statistical models ([9]). On the other hand, LAML assumes that logf(u|y,θ) is approximately quadratic as a function of *u* but no assumptions are made about the shape of logL(θ;y). An approximate quadratic shape for the likelihood is needed only if the LAML is used for computing confidence intervals based on the Fisher Information matrix or for conducting likelihood ratio test.

We consider linear and generalized linear regression models when covariates are measured with error. This is a rich and highly relevant class of hierarchical models ([32,33]). The R programs for these examples are available. We encourage readers to analyze these examples with their own choices of parameter values and sample sizes. For the LAML, we used the R package TMB ([24]). The R code for Monte Carlo (MC), Geyer–Thompson (GT), Data Cloning (DC) and Data Cloning followed by Data Doubling (DCDD) was developed by the authors and depends on the R packages for JAGS and dclone ([34,35,36]). In the figures, we use the term TMB to denote the LAML. We also conducted the usual convergence diagnostics for the MCMC-based methods as well as the diagnostics for the TMB. None of the diagnostics indicated any issues with the final results.

### 3.1. Single Parameter Models

We start with the case where only one parameter is unknown. In this case, we can use simple Monte Carlo methods to compute the true likelihood function accurately. This will be our ‘gold standard’ with which all other approximations are compared.

*Example 1(a): Normal linear regression through origin*

We assume that the response variables are $Y_{i}|X_{i}=x_{i}∼N\left(x_{i}\beta_{0},\sigma_{2}^{2}\right)$ where i=1,2,…,n. The observed covariates, measured with error, are $W_{i}|X_{i}=x_{i}∼N\left(x_{i},\sigma_{1}^{2}\right)$. The data consist of $\left(Y_{i},W_{i}\right)$ where i=1,2,…,n. For identifiability, we further assume that the distribution of the covariates is fully known. For the simulations, we assume $X_{i}∼T_{2}$, a T-distribution with 2 degrees of freedom. This is a relatively wide distribution.

**Figure 1:**
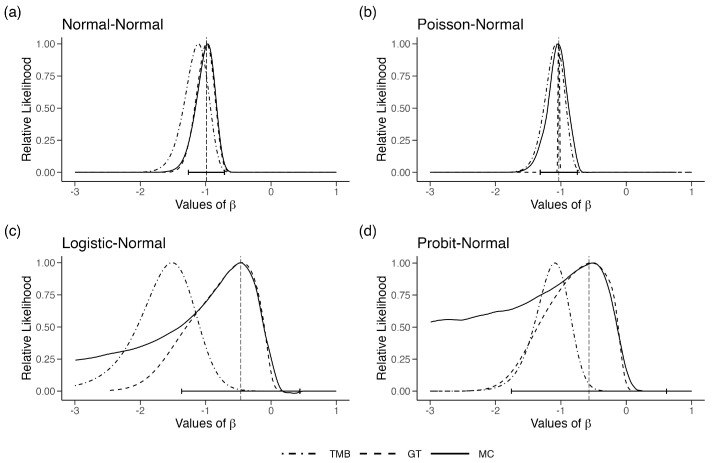
Relative likelihood for β in the four error-in-variable examples with one unknown parameter (single-parameter case). The vertical dashed line is the ML estimate for β and the horizontal error bars are their associated 95% CIs.

In Figure 1a, we plot the likelihood function obtained using MC, GT and TMB for a sample of size n=50 generated under the parameters $\left(\beta_{0}=-1,\sigma_{1}=3,\sigma_{2}=2\right)$. To begin, we assume $\sigma_{1}=3,\sigma_{2}=2$ are known and fixed. Hence, the only unknown parameter is $\beta_{0}$. The MC and GT likelihood functions are nearly identical to each other. The MLE of $\beta_{0}$, computed using DC, is also at the location of the maximum of the MC and the GT likelihood function. On the other hand, the LAML, using TMB, is substantially different than the true likelihood, reaching the maximum at a wrong location. This is the effect of the O(n) term mentioned in Equation (1), Section 2. Increasing the sample size does not correct for this bias. In fact, the confidence interval based on the LAML does not cover the true value as we increase the sample size.

*Example 1(b): Poisson regression through origin*

We wanted to see if the existence of both measurement error and environmental noise might be the reason for the behavior of the LAML function. We considered a somewhat different model where the responses are counts, an example of a GLMM with covariate measurement error. The response variables now are $Y_{i}|X_{i}=x_{i}∼Poisson\left(exp\left(x_{i}\beta_{0}\right)\right)$ where i=1,2,…,n. All other setup is as in Example 1(a).

In Figure 1b, we observe the same phenomenon that we observed with the normal regression model. The MC and GT likelihood functions match extremely well, but the TMB likelihood function is biased. The DC-MLE lies at the maximum of the MC likelihood function as it should.

*Example 1(c): Logit link binary regression through origin*

The difficulty of dealing with covariate measurement errors in Generalized Linear Models (GLM) is well known ([37]). Hence, we decided to change the model so that the response variables are extremely coarse, the binary responses. The response variables are $Y_{i}|X_{i}=x_{i}∼Binomial\left(1,p_{i}\right)$ where $p_{i}=\frac{exp(x_{i}\beta_{0})}{1+exp(x_{i}\beta_{0})}$ for i=1,2,…,n. All other setup is as in Example 1(a), except we increased the degrees of freedom to 10 for the distribution of X to make the likelihood function somewhat well behaved. Although simple in its description, this model structure is deceivingly difficult. In Figure 1c, we observe the same phenomenon that we observed with the Normal regression model. The MC and GT likelihood functions match somewhat reasonably, but the TMB likelihood function is quite biased. We note that, for some simulations, the GT likelihood function does not mimic the MC likelihood function, especially its curvature, very well. The DC-MLE lies at the maximum of the MC likelihood function.

*Example 1(d): Probit link binary regression through origin*

We decided to check the behavior under the Probit link function. The response variables are $Y_{i}|X_{i}=x_{i}∼Binomial\left(1,\Phi \left(x_{i}\beta_{0}\right)\right)$ where Φ(.) is the cumulative distribution function for a standard normal distribution and i=1,2,…,n. All other sets were the same as in Example 1(c). In Figure 1d, we plot the three likelihood functions. It is clear that the MC likelihood function is not a nice quadratic function. The DC-MLE is located at the maximum of the MC likelihood function. However, we needed a substantial number of clones to obtain the DC-based MLE. This is an indication of a substantial lack of information in the data. The GT-based likelihood function is highly dependent on the choice of the reference parameter. If we choose the reference parameter to be the DC-MLE with substantial effort, one can obtain the GT-based likelihood function. It does seem to conduct a better job than the LAML function. The LAML function is not only biased but is also highly concentrated with very narrow confidence intervals.

**Figure 2:**
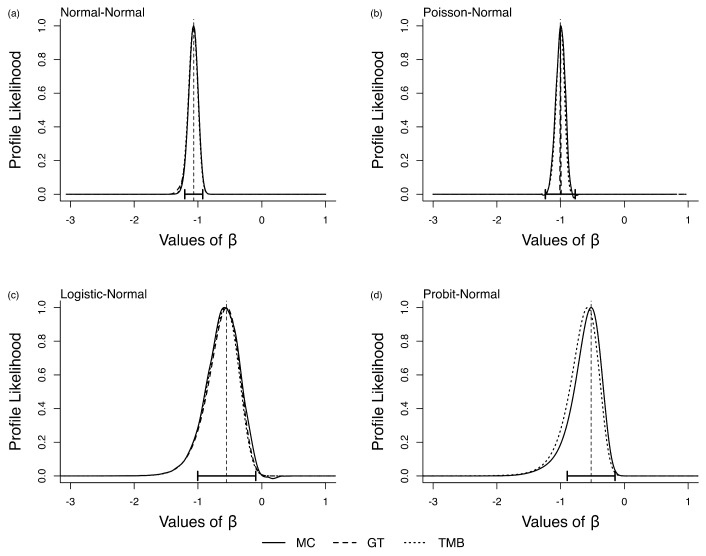
Relative likelihood for β in the four error-in-variable examples with one unknown parameter (single-parameter case), letting $\sigma_{1}$ and $\sigma_{2}$ in all cases equal to 1. The vertical dashed line is the ML estimate for β and the horizontal error bars are their associated 95% CIs.

We wanted to study if this behavior of LAML is due to some model structure inherent in the errors in variables models. To check this, we changed the parameter values to $\left(\beta_{0}=-1,\sigma_{1}=1,\sigma_{2}=1\right)$. Surprisingly, for this parameter combination, the MC and TMB match each other nearly perfectly. See Figure 2a–d. This is disturbing because this means the bias in LAML is not due to the model structure. If the bias in LAML were a function of the model structure, it would have been possible to provide guidance as to when it would be appropriate to use LAML in practice. On the other hand, because the bias depends on a particular parameter combination, that is, it works for some parameter combinations and not others, the issue is highly problematic; we can never be sure whether or not the statistical inferences deduced from a particular dataset are valid. As was noted by various discussants in Lee and Nelder (1996), the issue is whether or not the data in hand provide enough information about the latent variables. The answer depends both on the model structure and the true parameter values. In practice, true parameters are unknown and the answer to this question is unknowable.

**Figure 3:**
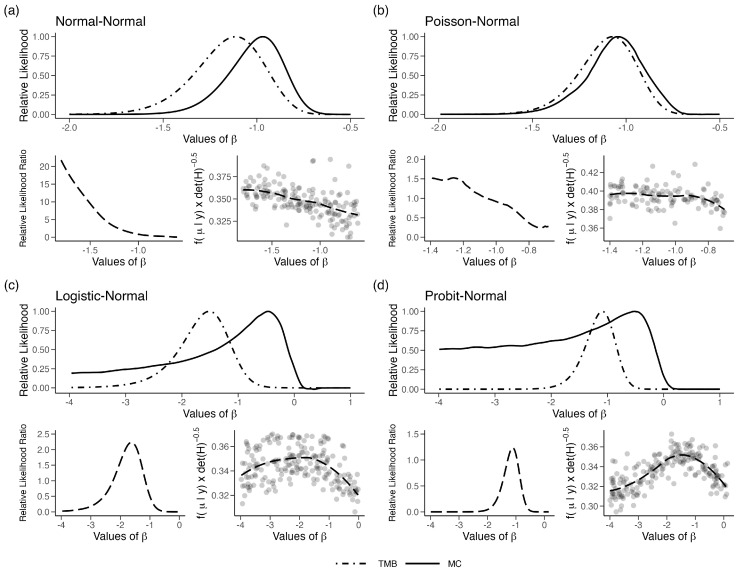
(Top plot in each panel) Relative likelihood for β in the four error-in-variable examples with one unknown parameter (single-parameter case). (bottom left in each panel) The relative likelihood, computed as the likelihood for LAML/TMB, plotted along with LOESS fit and (bottom right in each panel) $f\left({\hat{x}}_{1}|y_{1},w,\beta \right){\left|H\left({\hat{x}}_{1},y_{1},\beta \right)\right|}^{-0.5}$ plotted for different values of β. Points represent random draws for β from a uniform distribution.

To explore this further, we plotted $f\left(\hat{x}|y,w,\beta \right){\left|H\left(\hat{x},y,\beta \right)\right|}^{-0.5}$ (Equation (1), Section 2) for a single observation in the four cases where LAML failed. We simulated random numbers from $f(x_{1}|y_{1},w_{1},\beta )$ corresponding to the first sample $\left(y_{1},w_{1}\right)$ using the MCMC algorithm. Note that such random variates are also generated in the GT algorithm for approximating the likelihood function. Given these samples, we obtain an estimate of the density at the mode and an estimate of the curvature at the modal value using a non-parametric density estimator. Repeating these steps for different values of β, one can plot $f\left(\hat{x}|y,w,\beta \right){\left|H\left(\hat{x},y,\beta \right)\right|}^{-0.5}$ as a function of β. Recall that for the LAML to work well, $f\left({\hat{x}}_{1}|y_{1},w,\beta \right){\left|H\left(\hat{x_{1}},y_{1},\beta \right)\right|}^{-0.5}$ should be a constant, independent of the value of β. In Figure 3a–d, we plot this function for the four models described above. In the figure, we also plotted a LOESS fit for these points to smooth out the Monte Carlo variation. We first note that this bias function (Equation (1), Section 2), instead of being a constant, varies with β in all four cases. Moreover, the shape of this function mimics the ratio of the TMB based likelihood function and the MC-based likelihood function. This figure illustrates that the bias factor developed in (Equation (1), Section 2) is, indeed, correct. It is also somewhat surprising that, at least for one observation, the deviation of the bias function from a constant does not seem large, but its impact on the shape of the likelihood is substantial.

We also observed that if we change the distribution of *X* from a *T*-distribution with 2 degrees of freedom to 10 degrees of freedom, making it closer to a Gaussian distribution, the behavior of the LAML improves. Under this situation, especially under the Example 1(a) set up f(x|y,θ) is closer to a Gaussian distribution and the ‘bias’ factor or the penalty described in Section 2, Equation (1) is nearly 0 on the logarithmic scale. It is clear that the performance of the LAML strongly depends on the magnitude of the divergence of the conditional distribution of the latent variables, given the observations from a Gaussian distribution. This deviation could be large due to two factors. One, the information in the observations about the latent variables and also on the form of the marginal distribution f(u;θ) of the latent variables. When this marginal distribution is close to a Gaussian distribution, as in the built-in assumptions of INLA, the LAML is likely to work well. On the other hand, the MCMC-based methods are applicable in more general setups. The hierarchical models are popular and useful in many fields precisely because they can allow far more flexibility than standard Gaussian models ([20]). The wide use of LAML is probably undesirably shrinking the model space towards the Gaussian models.

### 3.2. Profile Likelihood and Multiparameter Models

We now consider the full parameter space for the models in Section 3.1. In this case, we are interested in studying the profile likelihood function for $\beta_{0}$ using GT ([12]), DCDD ([16]) and TMB ([24]). We wanted to study if computing the profile likelihood function exacerbates the bias observed in the single parameter models.

**Figure 4:**
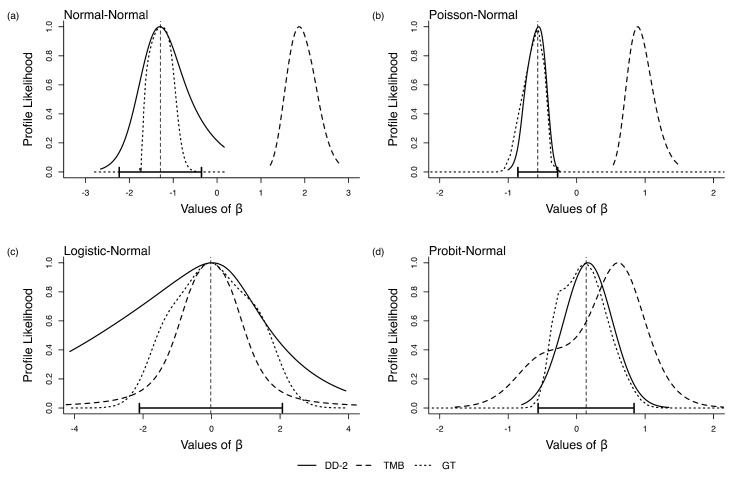
Profile likelihood for β in the four error-in-variable examples with no known parameters (multi-parameter case). The vertical dashed line is the ML estimate for β and the horizontal error bars are their associated 95% CI.

In Figure 4a–d, we present the results for the four models. We see a pattern similar to that we saw in the single parameter case; LAML is biased. A minimum requirement for the profile likelihood is that it is centered at the MLE of $\beta_{0}$. The GT and DCDD profile likelihoods have this feature by design because the DC-MLE and its asymptotic variance are used in computing the profile likelihood under DCDD and GT. The LAML profile likelihood, on the other hand, is clearly not centered on the MLE. As was the case in the single parameter models, LAML bias depends on the parameter combination and data in hand. LAML may give the right answer in some situations, but one would never know if the answer one has for the data in hand is, indeed, correct. We also note that the DCDD-based profile likelihoods are much more spread out than those based on LAML and GT.

### 3.3. Nonidentifiable Models

We now study how these methods work when the parameters are non-identifiable; a priori, we do not expect any of the methods to work. The question is as follows: Do they provide any diagnostics or ‘red flags’ for non-identifiability? In most practical situations, hierarchical models tend to be notoriously complex (e.g., [38,39]). Analytical assessment of parameter identifiability is nearly impossible for such models. However, it would be a major advantage if a method provided diagnostics for potential non-identifiability (e.g., [40]). The Bayesian approach based on MCMC provides such diagnosis either through non-convergence of the chains or lack of Bayesian learning ([41]). The method of data cloning has foundations in the Bayesian approach. It has built-in diagnostics for non-identifiability provided the set of non-identifiable parameters is a connected set ([14,42,43]). These diagnostics are based on the result that if the parameters are non-identifiable, as the sample size increases, the Bayesian posterior distribution converges to a non-degenerate distribution ([44]). Hence, as the sample size increases, the posterior variance converges to a non-zero quantity. In the case of data cloning, if the parameters are non-identifiable (strictly speaking, non-estimable), as the number of clones increases, the posterior variance converges to a non-zero quantity. This DC diagnostic tool works even for non-trivial phylogenetics models where the parameter is a tree topology ([45]). We note that data cloning cannot diagnose nonidentifiability reliably if the set of non-identifiable parameters is a disconnected set. If the set of non-identifiable parameters is a disconnected set, the likelihood function is a multimodal function with modes separated from each other by a valley. For such situations, most MCMC algorithms tend to get stuck in one or the other peak. Data cloning fails in such situations because it innately depends on the successful convergence of the MCMC algorithm.

*Example 5(a): Poisson regression with quadratic mean function and covariate measurement error*

**Figure 5:**
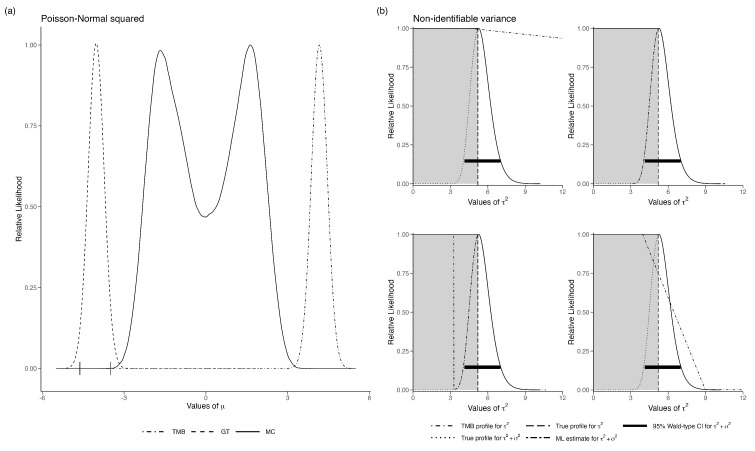
(**a**) Relative likelihood for β in the non-identifiable Poisson-Normal error in variables model with one unknown parameter. The horizontal error bars are the 95% CI for the data-cloned MLE. (**b**) Four examples of the relative likelihood for $\tau^{2}$ in the non-identifiable variance example. Grey shaded area is the range over which $\tau^{2}$ should be flat, i.e., the sum of $\tau^{2}$ and $\sigma^{2}$. Each plot in (**b**) represents a different iteration of the model using the same data.

In the following, we show that GT and LAML fail when the likelihood modes are well separated. Let $Y_{i}|X_{i}=x_{i}∼Poisson\left(x_{i}^{2}\right)$ and $X_{i}∼N\left(\beta ,5\right)$ where i=1,2,…,n. It is obvious that the observed data $Y_{i}$ cannot provide unambiguous information about the value of $X_{i}$. This is reflected in the bimodality of the MC likelihood function for β in Figure 5a. As expected, the DC-MLE converges to one of the modes. When using data cloning, it is suggested that many different priors be used. If they all converge to the same point ([13]) then we can rely on the results. In this particular example, we started the priors with means 5 and −5, and they both converged at the same point. Clearly, data cloning cannot diagnose the non-identifiability problem reliably when the sets are disjointed. To our surprise, the GT-based likelihood also failed. We tried using different reference points, and they all converged to the same estimate of the likelihood function; however, it was the wrong one. Similarly, the LAML computation indicated no issues with the model and gave a nice-looking, albeit completely incorrect, likelihood function. It seems that if the non-identifiable parameter set is a disjointed set, none of the methods work. Even worse, all of them give wrong answers quite confidently.

*Example 5(b): Measurement error model with no replication*

We now consider the case where the set of non-identifiable parameters is connected. Let $Y_{i}|\mu_{i}∼N\left(\mu_{i},\sigma^{2}\right)$ and $\mu_{i}∼N\left(\mu ,\tau^{2}\right)$ for i=1,2,…,n. For this model, the marginal distribution of the observed data is $Y_{i}∼N\left(\mu ,\sigma^{2}+\tau^{2}\right)$. Clearly, we cannot identify $\sigma^{2}$ and $\tau^{2}$ separately, although $\sigma^{2}+\tau^{2}$ is identifiable. The likelihood function has a ridge instead of distinct peaks.

This example was studied by [42]. See also [46] for application in a population dynamics models with observation error. The MCMC algorithm, especially with flat priors, does not converge, raising a red flag. With highly informative priors, the MCMC algorithm can be made to converge. Such informative priors can be used to obtain the MLE using data cloning. Thus, MCMC convergence issues can be potentially surmounted. However, it was shown in [42] that as the number of clones increases, the bivariate posterior distribution of $\left(\sigma^{2},\tau^{2}\right)$ converges to a non-degenerate distribution concentrated on a diagonal and its variance does not converge to zero.

We applied LAML to this situation. The LAML behaves quite differently. For the same dataset, if we provide different starting values for the maximization with respect to the latent variables *u*, we obtain different likelihood functions. In Figure 5b, we show the marginal likelihood estimates for the same data but with several different starting values. For one set of starting values, the likelihood is nearly flat on the appropriate range; for another set of starting values, it is flat but on a wrong range and for another set of starting values, the likelihood function looks like a nice, quadratic function with mode at the sum of the two variances. Essentially, it converges to the model with no measurement error. For another starting value, it looks like a combination of a flat likelihood and the quadratic likelihood for the sum of the variances. Disconcertingly, LAML does not flag any of them to be wrong.

This example suggests that, when using LAML, the user should at least try several different starting values when optimizing with respect to *u* and check if they all provide similar likelihoods. If they do not, perhaps one should not use the LAML for inference. Although this is true in any numerical optimization routine, convergence to the same LAML is no guarantee that it is a good approximation. The number of different starting values and their locations in the domain of *U* will affect such diagnostics, especially given the high dimension of *U*.

### 3.4. Coverage Probabilities Using LAML

In the discussion above, we have shown the difference between the true likelihood and profile likelihood and the corresponding LAML approximation. By necessity, these differences are illustrated on a single dataset. Is it possible that we happened to come across a bad dataset by chance? To remove this possibility, we generated 500 datasets under the same models that we used earlier. If the bad approximations we observed were a result of a few rogue datasets, the coverage probabilities for confidence intervals based on the MC-based likelihood functions and the LAML-based confidence intervals would be nearly equal. As we show, this is not the case. The coverage probabilities are substantially different, again supporting our claim that LAML can be misleading.

**Figure 6:**
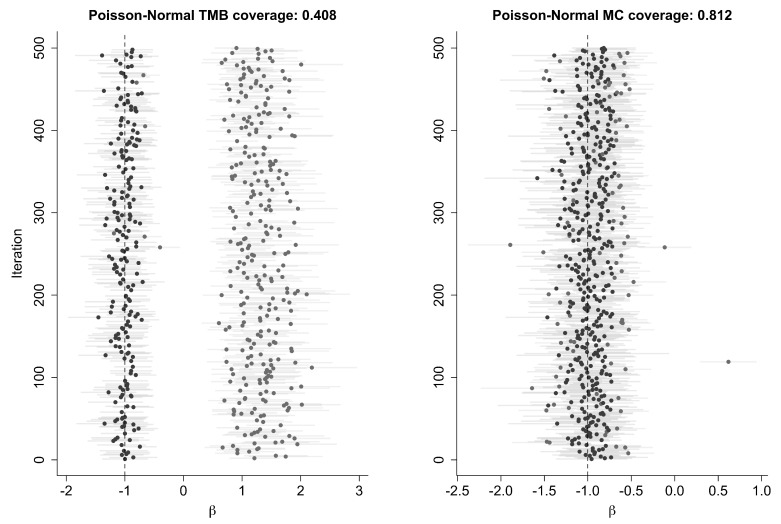
Coverage results for the Poisson-Normal error-in-variables model using TMB and MC. Points represent the estimate for β from 500 models, with error bars showing the 95% CI. Intervals including the true value for β at −1 are colored black; those not including the true value are grey.

The Poisson-Normal model here is the identifiable model described in Example 1(b). The reason that one obtains such bad coverage using LAML seems to stem from the multimodality of f(u|y,θ) with respect to *u*. In some cases, the numerical maximization routine finds the wrong model. We illustrate this with a plot of 500 confidence intervals for this example in Table 1 and Figure 6. Clearly, there are two sets of confidence intervals, one near −1 and one near +1.

## 4. Discussion

Although hierarchical models are extremely useful to model complex phenomena, conducting statistical inference for them is difficult. The MCMC algorithms are useful computational tools towards that goal. These algorithms, being computationally intensive, are unattractive for analyzing large datasets or complex models ([6]). An alternative to using the MCMC algorithms is based on the Laplace approximation to the conditional distribution of the latent variables given the observed data. This approach is significantly faster than the MCMC algorithm and hence has been used widely in applications. The failure of this approach, as illustrated in this paper, is related to the fact that the conditional distribution of the latent variables is not always well approximated by a Laplace approximation. We also showed that the failure of the Laplace approximation is not a function of the model structure. For the same model structure, for some parameters, the approximation may work well, but for other parameters, it could fail. This makes it difficult to provide a general recommendation on when one can use Laplace approximation to the marginal likelihood. This approximation also depends strongly on the assumption that the conditional distribution of the latent variables, given the observations, is unimodal. In many hierarchical models, the dimension of the latent variables is larger than the dimension of the observed data. For such models, it is highly unlikely that f(u|Y=y,θ) is unimodal for all θ and for all samples. Even when the mode is unique, there is no guarantee that this distribution is quadratic near the mode for all hierarchical models. The main problem with the Laplace approximation seems to be that, in many situations, one cannot replace the averaging operation by maximization. Given these caveats and counter examples, we suggest that LAML should be used for statistical inference only with significant care and caution.

The Achilles heel for the MCMC-based approaches is their substantial computational burden. On the other hand, the Achilles heel for the Laplace approximation of the marginal likelihood is the possible lack of information in the observations about the latent variables. It is impossible to increase information in the observations, but perhaps we can use the Laplace approximation to point us to an appropriate subset of the parameter space where the MLE might be located. This good set of starting values can then be used to speed up the general purpose optimization methods, such as Data cloning (DC), Monte Carlo Newton Raphson (MCNR) or Monte Carlo Expectation Maximization (MCEM) for conducting statistical inference.

## Figures and Tables

**Table 1:** Coverage results for β using LAML and MC.

Model	Model Parameters	Inference Parameter	MC Coverage	LAML Coverage
Normal-Normal	β=−1, $\sigma_{1}=3$, $\sigma_{2}=2$, df=2	β	0.932	0.838
Normal-Normal	β=−1, $\sigma_{1}=3$, $\sigma_{2}=2$, df=3	β	0.946	0.846
Logit-Normal	β=−1, $\sigma_{1}=3$, df=10	β	0.995	0.880
Probit-Normal	β=−1, $\sigma_{1}=3$, df=10	β	0.900	0.908
Poisson-Normal	β=−1, $\sigma_{1}=3,$df=2	β	0.836	0.384
Poisson-Normal	β=−1, $\sigma_{1}=3,$df=3	β	0.812	0.408

## Data Availability

The code is available at Available online: https://github.com/jmponciano/TestingLaplace (accessed on 22 December 2024).
